# Analysis of multiple organ damage and clinical immunological characteristics in systemic lupus erythematosus patients with hematologic involvement

**DOI:** 10.7150/ijms.48997

**Published:** 2021-05-01

**Authors:** Liming Tan, Yonglei Zhao

**Affiliations:** 1 Department of Clinical Laboratory, Second Affiliated Hospital of Nanchang University, Jiangxi Key Laboratory of Laboratory Medicine, Nanchang 330006, China.; 2 Second Clinical Medical College, Nanchang University, Nanchang 330006, China.

**Keywords:** systemic lupus erythematosus, hematological involvement, immunological characteristics, imaging findings

## Abstract

**Objective:** To investigate clinical immunological characteristics and imaging findings of multiple organ damage of systemic lupus erythematosus (SLE) patients with hematologic involvement.

**Methods:** SLE patients diagnosed in the Second Affiliated Hospital of Nanchang University from June 2015 to March 2019 were selected, including 93 SLE patients with hematologic involvement and 68 SLE patients without hematologic involvement. Immunological indicators such as autoantibodies, immunoglobulin G (IgG), complement 4 (C4) and imaging data of several organs were measured respectively. The results were statistically analyzed.

**Results:** SLE patients with hematologic involvement were more likely to have autoimmune hemolytic anemia (AIHA) (20.43%, *P*<0.05). The erythrocyte sedimentation rate (ESR) of SLE patients with hematologic involvement was 75.82 (±35.33) mm/h, IgG was 28.84 (±6.00) g/L and C4 was 0.073 (±0.031) g/L (*P*< 0.05). The area under the curve (AUC) of IgG was the highest among the above indicators (*P*<0.01). The positive anti-RO-52 antibody (OR=15.926, *P*<0.05) was an independent risk factor for pulmonary inflammatory lesions in SLE patients with hematologic involvement.

**Conclusion:** Compared with the control group, abnormal immunological indicators and multiple organs damage are more obvious. Positive anti-RO-52 antibody may play an important role in the pathogenesis of pulmonary inflammation in SLE patients.

## Introduction

Systemic lupus erythematosus (SLE) is an autoimmune disease that affects a variety of systems and organs and is more common in women. Studies have shown 1, 2 that the kidney is one of the most easily affected organs in SLE. In recent years, other organ and system damages of SLE patients have been gradually reported, such as blood system, nervous system damages and gastrointestinal dysfunction 3-5. Studies have shown that some patients' blood system has been injured to varying degrees, including thrombocytopenia, anemia, leukopenia and other clinical features, and combined with damage to vital organs such as liver, heart and lung 5, 6. Enhanced immune system activity is one of the main manifestations of SLE, which are characterized by high gamma globulinemia, immune complex formation and activation of the complement system. The core of its pathogenesis is the highly specific reaction between autoantibodies and autoantigens. These reactions interact with each other in the blood system circulation in SLE patients, causing early damage of some organs. Clinical workers may miss these organ damage. This study analyzed imaging characteristics of hematologic abnormalities in autoantibodies, immunoglobulin, complement, and organs (such as the heart and lungs) of SLE patients. According to these indicators, this study provides a reference basis for correctly determining the outcome of SLE clinically and adopting a more comprehensive and timely treatment regimen.

## Methods

### Patients

93 SLE patients with hematologic involvement and 68 SLE patients without hematologic involvement diagnosed in the second affiliated hospital of Nanchang university from June 2015 to March 2019 were selected. All patients met the standard (SLICC) formulated by SLE international cooperative clinic in 2012 7. Diagnostic criteria for abnormal hematologic manifestations include: leukopenia (whole blood leukocyte count < 4×10^9^/L); Autoimmune hemolytic anemia (AIHA) (1 hemoglobin level reaches anemia standard. 2 erythrocyte autoantibodies are detected. 3 at least one of the following: reticulocyte percentage > 4% or absolute value > 20×10^9^/L; combined globin <100 mg / L; total bilirubin ≥ 17.1 μ mol / L, with unbound bilirubin increased); thrombocytopenia (platelets <100×10^9^/L). All specimens were collected with the informed consent of the patients. The study was approved by the Ethics Committee of the Second Affiliated Hospital of Nanchang University.

### Inclusion criteria

All participants met the following criteria: 1) signed informed consent, voluntary participation, 2) with clear diagnosis, complete clinical data and imaging data, 3) Any drugs that seriously affect the blood system, heart and lungs were not taken within three months of admission.

### Exclusion criteria

Exclusion criteria were as follows: 1) patients with other autoimmune diseases; 2) patients with severe infectious diseases or other causes of hematological diseases within 3 months; 3) patients with malignant tumors; 4) pregnant or lactating patients; 5) lupus caused by drugs.

### Instruments and methods

IgG, C3 and C4 were measured by Beckman Immage800 specific protein analysis system and associated detection reagent (Beckman, USA). Erythrocyte sedimentation rate (ESR) measured by Percil XC - 40 full-automatic dynamic blood sedimentation. Indirect immune fluoresecence (IIF) was used to measure antinuclear antibody (ANA) and anti-double-stranded DNA (ds-DNA) antibody. The anti-nuclear antibody spectrum assay kit, EURO Blot Master II and linear immunoblot assay (LIA) were used to measure the anti-nuclear antibody spectrum. CT examination was used by GE 64-slice Light Speed VCT, (GE Healthcare), Philips 16-slice spiral CT (Philips Healthcare) and Siemens Somatom Definition Flash (Siemens Healthcare) for axial or coronal scanning of vital organs such as lungs and liver. Philips IE33 type and Philips IE elite type multifunctional color doppler ultrasound diagnostic instrument were used with a frequency of 1.0 ~ 5.0 mhz. The patients were placed in left lateral position and supine position to examine the heart, pericardium, liver and spleen. All operations were carried out in strict accordance with the instructions of each reagent and the quality management standard documents of the second affiliated hospital of Nanchang university.

### Statistical analysis

Statistical analysis of the data was performed by SPSS program version 22. Continuous variables of normal distribution were expressed as mean ± standard deviation (x±s), and T-test was adopted for comparison between any two means. Continuous variables of non-normal distribution was described by M (P25, P75), Mann-Whitney U test was used for comparison between any two means. Chi-square test and Fisher's exact test were used for categorical variables. A binary logistic regression model was used to analyze the independent risk factors for each organ injury. Receiver operating characteristics (ROC) analysis was performed, and the best cut-off value was determined. Area under the curve (AUC) was calculated. AUC=0.5~0.7 indicated low diagnostic accuracy. AUC=0.7~0.9 indicated a certain diagnostic accuracy. AUC > 0.9 indicated a high diagnostic accuracy. *P*<0.05 was considered statistically significant.

## Results

### Basic characteristics of SLE patients with and without hematologic involvement

Compared with SLE patients without hematologic involvement, SLE patients with hematologic involvement were more likely to have AIHA (*P* < 0.05) and accelerated ESR (*P* < 0.01). As shown in **Table 1**.

### Immunological characteristics of SLE patients with hematologic involvement and SLE patients without hematologic involvement

Compared with SLE patients without hematologic involvement, SLE patients with hematologic involvement had higher IgG and quantitative anti-dsDNA antibodies than patients without hematologic involvement. The levels of complement C3 and C4 in SLE patients with hematologic involvement were lower, (0.45±0.19g/L and 0.073±0.031 g/L) respectively (*P*<0.01). The positive rate of anti-SS-B and anti-AnuA was higher (*P* < 0.05). As shown in **Table 2**.

### ROC curve analysis of ESR and immunological indicators

**Figure 1:**
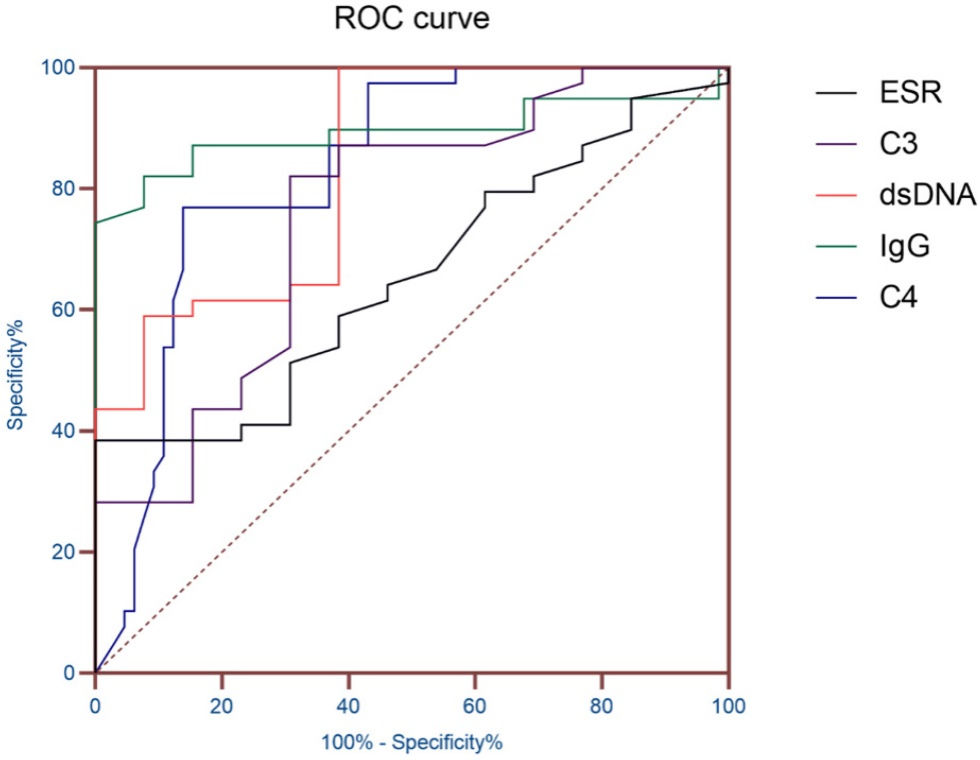
ROC curve of ESR and Immunological indicators.

AUC (95% CI) was obtained according to ESR, dsDNA, IgG, C3 and C4, so as to evaluate the diagnostic value of these indicators in SLE patients with hematologic involvement. The results showed that the AUC of ESR, dsDNA, IgG, C3 and C4 were all greater than 0.7, and the AUC of IgG was 0.891 (*P*< 0.01), which proved that it has good diagnostic value for SLE hematological system involvement (**Table 3 and Figure 1**).

### The imaging findings of lung, liver and other vital organs in the two groups

CT and ultrasonography showed that SLE patients with hematologic involvement were more likely to have pulmonary inflammatory lesions, hepatic cysts and splenomegaly (*P*<0.05). As shown in **Table 4**.

### Binary Logistic regression analysis of pulmonary inflammatory lesions

To further investigate whether the imaging features of these organ lesions were related to autoantibodies, a multivariate binary logistic regression model was used in this study. Due to the word limit, only a few items are selected and listed in **Table 5**. The results showed that RO-52 antibody positive was an independent risk factor for pulmonary inflammatory lesions after exclusion of confounding factors (OR=15.926, P<0.05).

## Discussion

SLE patients had diverse immunological characteristics and clinical manifestations. In previous studies, scholars mainly focused on LN, which is a kind of common and severe organ damage caused by SLE 2, 8. In recent years, a number of studies have shown that an increasing number of systems and organs were involved in SLE besides kidney, including blood system, nervous system and gastrointestinal tract, which may be caused by the formation of immune complex, complement system activation and specific autoantigen antibody reaction in SLE patients 4, 9, 10. This study is the clinical data of patients with SLE blood system involvement. This retrospective analysis was conducted to explore the characteristics of blood system involvement in SLE patients and observe the relationship between blood system involvement and SLE Clinical indicators, laboratory indicators to guide clinical diagnosis and treatment.

The results showed that the gender and age distribution of the two groups were similar. The hematologic involvement in SLE patients is mainly characterized by AIHA, leukopenia, and thrombocytopenia, consistent with a retrospective analysis of Anum Fayyaz 6, which were common manifestations of hematologic lesions in SLE. SLE patients with hematologic involvement were more likely to have abnormal blood system manifestations. Compared with a study conducted by El, H.K. et al in a clinic in Cairo, Egypt 11, the probability of leukopenia in this study was lower, which may be caused by different geographical, ethnic and diagnostic levels. Then, ESR and immunological indicators of the patients were analyzed. Compared with SLE patients without hematologic involvement, patients with hematologic involvement had higher IgG and quantitative anti-dsDNA antibodies than patients without hematologic involvement. Compared with SLE patients without hematologic involvement, the levels of complement C3 and C4 in SLE patients with hematologic involvement were lower. Previous studies 12, 13 have shown that IgG, C3 and C4 can be used as indicators to monitor the active stage of SLE disease, and hematologic involvement in the clinical diagnostic guidelines of SLE disease was also classified as the performance of SLE disease activity. The results of this study were proved to be reliable. The ANA and ENAs were analyzed. The results of the autoantibody study in SLE patients were similar to the study by Feng, X. et al. 15 in Meizhou, Guangdong Province, China. And the positive rates anti-dsDNA antibody were also basically consistent. It was noteworthy that in the analysis of ENAs, the anti-SS-B antibody of SLE patients with hematologic involvement showed a higher positive rate of 26.88%, significantly higher than that of the other group (13.24%). In previous studies 16, 17, anti-ANuA antibodies were considered to be a specific antibody besides dsDNA in SLE patients with specificity of up to 90% and were associated with disease activity in SLE. It was consistent with this study. Anti-dsDNA antibody is one of the self-specific antibodies of SLE, the positive rate of which reached 48.39% in this study. In a study conducted by Gheita, T.A. et al. 18, anti-dsDNA antibody titers were associated with ESR, and dsDNA quantitative analysis was used in this study to make the correlation between the indicators more intuitive and accurate. Finally, AUC (95% CI) was obtained according to ESR, dsDNA, IgG, C3 and C4, so as to evaluate the diagnostic value of these indicators in SLE patients with hematologic involvement. The results showed that the AUC of IgG was 0.891, which proved that it has good diagnostic value for SLE hematological system involvement.

In addition, this study analyzed the imaging characteristics of organs in the two groups of patients. The imaging features of the lungs were mainly inflammatory lesions, small nodules, consolidation and cord lesions. The positive rate of pneumonia lesions in SLE patients with hematologic involvement was 69.89%, which was higher than that of the other group (51.47%). The lungs involvement of SLE has been reported in the past 19-21, the probability of lung involvement is lower than the results in this study, which may be caused by smoking, treatment levels ,individual immunity and other factors. The imaging features of the heart were mainly valvular regurgitation (mainly mitral, aortic, tricuspid), left ventricular diastolic dysfunction, pericardial effusion, although the difference between the two groups was not statistically significant, but both had a high positive rate which suggested that there may be damage to the heart in SLE patients in the early stages, especially the valve damage. In previous studies, it has been suggested that the heart of SLE patients is very vulnerable 22, 23, so it must be taken seriously. In addition, liver and spleen were also easily affected by SLE. The results showed that hepatic cysts, hepatic calcification, fatty liver and splenomegaly were common imaging findings in SLE patients, and SLE patients with hematologic involvement more likely had hepatic cysts and splenomegaly. As mentioned above, organ damage in SLE patients with hematologic involvement were very common, which may be caused by the specific antibodies and complement of SLE patients as blood circulated in various organs and eventually reacted and accumulated. A multivariate binary logistic regression model was used to investigate whether the imaging features of these organ lesions were related to autoantibodies in this study. The results showed that RO-52 antibody positive was an independent risk factor for pulmonary inflammatory lesions after exclusion of confounding factors. For SLE patients with positive RO-52 antibody, patients should be closely monitored and paid attention to the lungs of patients to reduce the incidence of lung inflammation and improve the prognosis of the disease. In the process of diagnosis and treatment, it was necessary to review and closely monitor the functions of vital organs such as liver and lung to prevent irreversible damage. In addition to multiple organ damage in patients caused by SLE disease itself, in the treatment of SLE disease, a large number of glucocorticoids and biological agents need to be used for patients, which may also cause chronic failure of many organs of patients 24, 25. Therefore, attention must be paid to the monitoring and treatment of multiple organ functions in SLE patients.

In conclusion, it was proved that SLE patients with hematologic involvement were more likely to have abnormal immunological characteristics. Heart, lung and liver damage were more likely to occur in these patients. The positive anti-RO-52 antibody may promote the occurrence of pulmonary inflammation. It was suggested that the damage of various organs and systems in SLE patients should be diagnosed early to reduce the occurrence of systemic lupus erythematosus involving the blood system thereby improving the prognosis of patients.

## Figures and Tables

**Table 1:** Basic characteristics of the two groups of patients

	SLE with hematologic involvement (N = 93)	SLE without hematologic involvement (N = 68)	*P* value
Female	83 (89.25)	62 (91.18)	0.686
Age (years)	38.7±16.4	40.8±14.4	0.434
AIHA	19 (20.43)	6 (8.82)	0.045
Leukopenia	12 (12.90)	11 (16.17)	0.558
Thrombocytopenia	38 (40.86)	21 (30.88)	0.194
ESR (mm/h)	75.82±35.33	47.79±24.58	0.001

Note: T-test and Chi-square test were used. SLE, systemic lupus erythematosus; AIHA, autoimmune hemolytic anemia; ESR, erythrocyte sedimentation rate.

**Table 2:** Immunological characteristics of the two groups' patients

	SLE with hematologic involvement (N = 93)	SLE without hematologic involvement (N = 68)	*P* value
C3 (g/L)	0.45±0.19	0.65±0.21	<0.001
C4 (g/L)	0.078±0.029	0.136±0.053	<0.001
IgG (g/L)	28.84±6.00	21.49±3.56	<0.001
Anti-dsDNA (IU/ml)	174.60 [(87.61,643.80)]	36.28 [(29.04,126.79)]	<0.001
ANA	90 (96.77)	66 (97.06)	>0.999
Anti-dsDNA	45 (48.39)	32 (47.06)	0.868
Anti-SS-A	54 (58.06)	38 (55.88)	0.782
Anti-SS-B	25 (26.88)	9 (13.24)	0.036
Anti-Ro-52	47 (50.54)	30 (44.18)	0.421
Anti-Sm	19 (20.43)	16 (23.53)	0.638
Anti-Histone	17 (18.28)	14 (20.59)	0.714
Anti-ANuA	56 (60.22)	29 (42.65)	0.027
Anti-Rib P	22 (23.66)	18 (26.47)	0.683
Anti-nRNP/Sm	23 (24.73)	23 (33.82)	0.207
Anti-CENP-B	5 (5.38)	3 (4.41)	>0.999

Note: T-test, Chi-square test and Mann-Whitney U test were used. SLE, systemic lupus erythematosus; C3, Complement 3; C4, Complement 4; IgG, immunoglobulin G; ANA, anti-nuclear antibody; Anti-dsDNA, anti-double-stranded DNA; Anti-SS-A, anti-Sjogren's syndrome antigen A; Anti-SS-B, anti-Sjogren's syndrome antigen B; Anti-Sm, anti-Smith; Anti-Histone, anti-histone antibody; Anti-ANuA, anti-nucleosome antibodies; Anti-Rib P, Anti-ribosomal antibody.

**Table 3:** ROC curve analysis of ESR and immunological indicators

index	AUC	Standard error	*P* value	95% confidence interval
ESR	0.726	0.057	0.01	0.615-0.873
IgG	0.891	0.043	<0.01	0.806-0.975
C3	0.753	0.055	<0.01	0.645-0.861
C4	0.832	0.040	<0.01	0.753-0.910
Anti-dsDNA	0.826	0.064	<0.01	0.701-0.952

Note: AUC, Area under the curve; ESR, erythrocyte sedimentation rate; IgG, immunoglobulin G; C3, Complement 3; C4, Complement 4; Anti-dsDNA, anti-double-stranded DNA.

**Table 4:** Imaging findings of important organ lesions in the two group

Imaging findings	SLE with hematologic involvement (N = 93)	SLE without hematologic involvement (N = 68)	*P* value
**Chest/Lung**			
Inflammatory lesion	65 (69.89)	35 (51.47)	0.017
Small nodule	7 (7.53)	3 (4.41)	0.632
Strip stove	21 (22.58)	9 (13.24)	0.133
Solid change	21 (22.58)	14 (20.59)	0.762
lymphadenopathy	24 (25.81)	12 (17.65)	0.220
Pleural effusion	31 (33.30)	20 (29.41)	0.597
**Liver/Spleen**			
Hepatic cyst	28 (30.11)	10 (14.71)	0.023
Hepatic calcification	17 (18.28)	7 (10.29)	0.160
Fatty liver	9 (9.68)	1 (1.47)	0.072
Splenomegaly	12 (12.90)	3 (4.33)	0.045
**Heart**			
Valvular regurgitation	45 (48.39)	26 (38.24)	0.200
Left ventricular diastolic dysfunction	30 (32.26)	19 (27.94)	0.557
Pericardial effusion	24 (25.81)	23 (33.82)	0.269

Note: Chi-square test and Fisher's exact test were used.

**Table 5:** Binary Logistic regression analysis of pulmonary inflammatory lesions

Indicators	β value	SE	Wald	*P* value	Exp(B)	95% confidence interval
Anti-RO-52	2.768	1.117	6.140	0.013	15.926	1.784, 142.21
Anti-SS-A	1.015	1.058	.920	0.337	2.759	0.347, 21.946
Anti-SS-B	-1.531	1.142	1.798	0.180	0.216	0.023, 2.027
Anti-dsDNA	0.002	0.001	2.467	0.116	1.002	1.000, 1.004

Note: Anti-SS-A, anti-Sjogren's syndrome antigen A; Anti-SS-B, anti-Sjogren's syndrome antigen B; Anti-dsDNA, anti-double-stranded DNA.
